# Genome-Wide Identification of the Gustatory Receptor Gene Family of the Invasive Pest, Red Palm Weevil, *Rhynchophorus ferrugineus* (Olivier, 1790)

**DOI:** 10.3390/insects12070611

**Published:** 2021-07-05

**Authors:** Patamarerk Engsontia, Chutamas Satasook

**Affiliations:** 1 Division of Biological Science, Faculty of Science, Prince of Songkla University, Songkhla 90110, Thailand; 2 Princess Maha Chakri Sirindhorn Natural History Museum, Prince of Songkla University, Songkhla 90110, Thailand; chutamas.p@psu.ac.th

**Keywords:** chemoreception, gene annotation, gustation, insect taste receptor, oil palm pest

## Abstract

**Simple Summary:**

The red palm weevil (*Rhynchophorus ferrugineus*) is an invasive pest that causes severe economic loss in palm plant cultivation (e.g., oil palm, date, and coconut) in many regions of the world. The development of efficient approaches to controlling this pest is urgently needed. Previous research focused on the molecular basis of its olfaction, leading to the identification of this species’ aggregation pheromone receptor, which can help develop biosensors and bait traps. To gain more understanding of the chemoreception in this species, we used a bioinformatic approach to identify all the gustatory receptor genes from its genome and transcriptome data. There are 50 gustatory receptor genes encoding 65 functional receptors, including CO_2_, sugar, and bitter receptors. Our results will help elucidate how this beetle discriminates and evaluates food and egg-laying sites and communicates via gustation.

**Abstract:**

The red palm weevil (*Rhynchophorus ferrugineus*) is a highly destructive pest of oil palm, date, and coconut in many parts of Asia, Europe, and Africa. The Food and Agriculture Organization of the United Nations has called for international collaboration to develop a multidisciplinary strategy to control this invasive pest. Previous research focused on the molecular basis of chemoreception in this species, particularly olfaction, to develop biosensors for early detection and more effective bait traps for mass trapping. However, the molecular basis of gustation, which plays an essential role in discriminating food and egg-laying sites and chemical communication in this species, is limited because its complete gustatory receptor gene family still has not been characterized. We manually annotated the gene family from the recently available genome and transcriptome data and reported 50 gustatory receptor genes encoding 65 gustatory receptors, including 7 carbon dioxide, 9 sugar, and 49 bitter receptors. This study provides a platform for future functional analysis and comparative chemosensory study. A better understanding of gustation will improve our understanding of this species’ complex chemoreception, which is an important step toward developing more effective control methods.

## 1. Introduction

**Figure 1:**
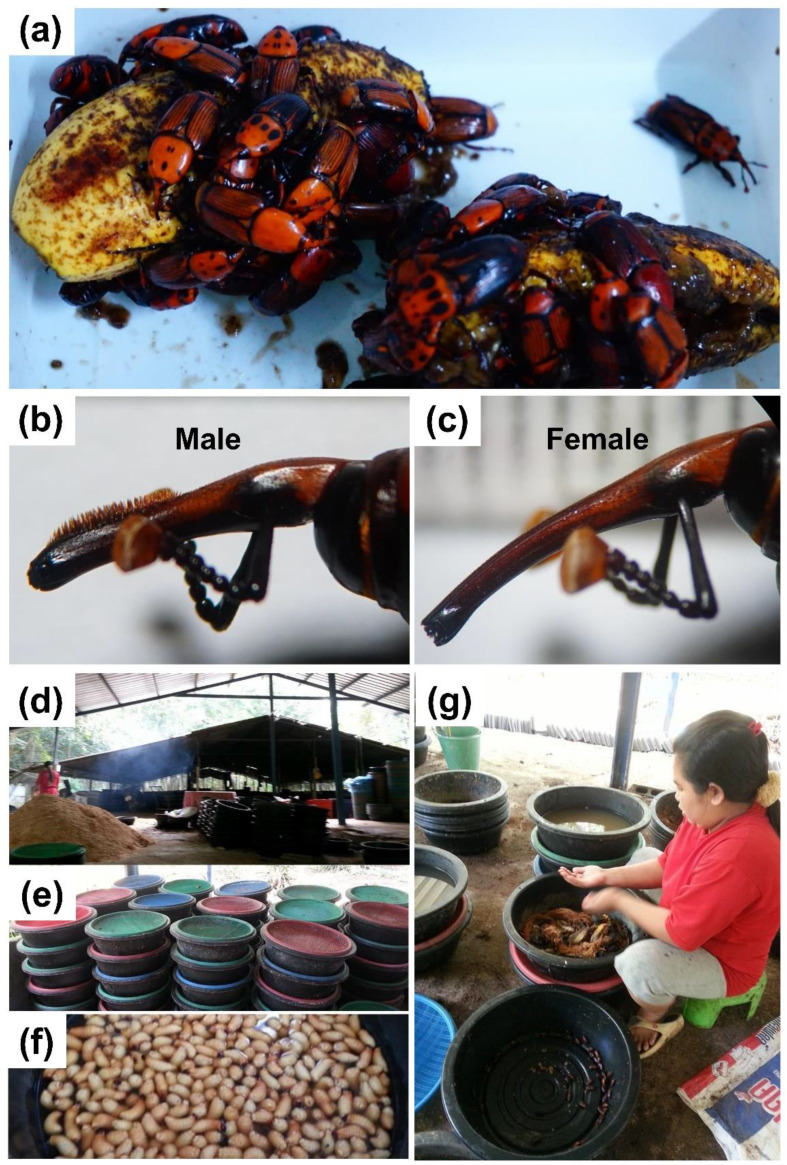
Red palm weevil (*Rhynchophorus ferrugineus*) (**a**) aggregating male and female adults (**b**) male mouthpart with chemosensory hairs (**c**) female mouthpart exhibiting sexual dimorphism (**d**–**g**) RPW farming (**d**) a local farm with a big pile of minced sago bark for rearing beetles (**e**) artificial nests for breeding beetles (**f**) larvae for sale as an exotic snack (**g**) a farmer is preparing adult beetles for breeding.

The red palm weevil (RPW), *Rhynchophorus ferrugineus* (Coleoptera: Curculionidae), is one of the world’s most invasive pest species, causing serious damage to palm plant cultivation worldwide [1,2]. It is a native species in Southeast Asia, but has dispersed to the Middle East, Europe, China, and North Africa, affecting the production of oil palms, coconuts, dates, and ornamental palms [1]. Female beetles infest trees and lay eggs inside the trunk, where larvae feed on the soft part and the apical growing part causing stunted growth and death, which in many cases can destroy the entire plantation [3]. The Food and Agriculture Organization (FAO) of the United Nations has declared RPWs a category-1 pest in the Middle East and North Africa and called for international collaboration and a multidisciplinary strategy to control this devastating pest [4]. Contrary to its profound negative impact, this beetle is an emerging economic insect in many countries, including Thailand. Their larvae, which are nutrient-rich, are bred in closed farms and sold as snacks (Figure 1d–g). A better understanding of its biology will help improve both control and breeding methods.

Chemoreception plays an essential role in the ecological success of RPWs, and some control methods rely on this sense. Male beetles produce an aggregation pheromone, consisting of (4RS, 5RS)-4-methylnonan-5-ol (ferrugineol) and 4(RS)-methylnonan-5-one (ferrugineone) that attracts both sexes [5,6,7]. This pheromone serves as a long-range attractant to direct beetles toward breeding grounds and colonized host plants [8]. In addition, the sternum cuticle of males and females contains 3-buten-2-ol and kopsinyl alcohol, respectively, which may be chemical cues that help beetles distinguish different sexes via gustation and olfaction [9]. Chemoreception also plays a vital role in finding correct plant hosts and appropriate egg-laying sites. Damaged or fermenting plant tissues also release chemicals that attract beetles [8]. Insect pheromones have long been used in integrated pest management programs. Traps with synthetic aggregation pheromones have been used to detect this pest early, before an outbreak; to lure-and-kill; and for mating disruption [10]. However, traps using an aggregation pheromone alone are not as effective as one using a pheromone blended with fermented plant tissues [8,11]. This suggests that chemical cues that can effectively attract this pest should be complex chemical blends rather than a single molecule. Thus, understanding the molecular basis of chemical attraction in RPWs requires knowledge about the entire receptor repertoire, olfaction, and gustation rather than a single receptor.

Advanced knowledge on the molecular basis of insect chemoreception can shed light on developing a super-effective pheromone bait or biosensors for pest control. Odorant receptors and gustatory receptors play essential roles in insect communication. Insect odorant receptors (ORs) are heterodimeric receptor complexes between a ligand-binding receptor and a conserved olfactory co-receptor (ORCO). Both types of receptor protein are about 350–400 amino acids in length and have seven transmembrane domains (TM), with an N-terminus inside the cell and a C terminus outside the cell [12,13]. The numbers of *Or* genes vary enormously among insects studied [14]. Some receptors are specifically sensitive to particular odorants (narrowly tuned), while others can be activated by a broader range of odorants (broadly-tuned) [15,16]. The *Orco* gene is highly conserved among neopteran insects [17]. It broadly expresses in every olfactory receptor neuron [18]. The ORCO protein has two essential roles: (1) it functions as an ion channel [19,20,21,22] and (2) as a chaperoning co-receptor forming a complex with ligand-binding ORs on the dendritic membrane [12]. Odorant receptors have been identified from the genome of some important beetle pests, including the red flour beetle *Tribolium castaneum* (264 ORs) [23], Colorado potato beetle *Leptinotarsa decemlineata* (75 ORs) [24], mountain pine beetle *Dendroctonus ponderosae* (86 ORs) [25], and Asian longhorned beetle *Anoplophora glabripennis* (120 ORs) [26].

Gustatory receptors (GRs) have a similar topology to ORs, and they share a common origin [27,28,29,30]. GRs respond primarily but are not limited to tastants. The signaling pathway occurs through the activation of ion channels [31,32] and G protein signaling pathways [33,34]. The number of *Gr* genes in the insect genome also greatly varies between species, possibly reflecting different ecological niches [35]. Gustatory receptors respond to many sweet and bitter tastants and other chemicals. The ability to discriminate the tastants allows insects to ingest only nutritious food and avoid toxic foods. In *Drosophila*, GRs senses bitter tastants, e.g., quinine, denatonium, berberine, lobeline papaverine, strychnine and L-canavanine [36,37,38], and sweet tastants (e.g., trehalose, melezitose, sucrose, maltose, and fructose) [39,40,41,42] have been identified. GRs also respond to other chemicals, e.g., glycerol [43] and CO_2_ [29,44,45,46]. Multiple studies suggest that GRs play essential roles in detecting cuticular hydrocarbons, which are chemical cues for social interaction and courtship. However, the functions of GRs as pheromone receptors have been demonstrated in *Drosophila* only [32,36,47,48,49]. Gustatory receptors have been identified from the genome of some important beetle pests, including *T. castaneum* (219 GRs) [50], *L. decemlineata* (144 GRs) [24], *D. ponderosae* (60 GRs) [25], and *A. glabripennis* (234 GRs) [26].

Odorant-binding proteins (OBPs) are small globular proteins (10–30 kDa or 90–270 amino acids) found in the sensilla lymph which share six conserved cysteine residues essential for forming globular shapes [51,52]. OBPs are involved in the solubilization of odorant molecules, predominantly hydrophobic molecules, and transport them to the odorant receptors on the dendritic membrane. The number of OBP genes in insect genomes varies from 5–83, reflecting their potentially diverse functions in ligand-binding specificity [53]. Some OBPs have an important role in sex pheromone reception, as reported in the silkmoth *Bombyx mori*, beetle, and *Drosophila* [52,54,55,56,57]. Odorant binding proteins have been identified from genomes of some important beetle pests, including *T. castaneum* (49 OBPs) [50], *L. decemlineata* (58 OBPs) [24], *D. ponderosae* (36 OBPs) [25], *A. glabripennis* (52 OBPs) [26].

Knowledge of the molecular basis of chemoreception in the RPW has advanced rapidly, particularly olfaction. Previous antennal transcriptome analyses identified some odorant receptor genes (18–77 genes), gustatory receptor genes (9–15 genes), and odorant binding protein genes (11–38 genes) [58,59,60]. Knocking down the *Orco* gene or *RferOBP1768* gene in the adult RPW using RNA interference (RNAi) reduces the beetles’ electrophysiological and behavioral responses to ferrugineol [61,62]. Recently, the odorant receptor RferOR1 was identified as an aggregation pheromone receptor. This gene is highly expressed in both male and female antennae. Its expression level across life cycles correlates with the reproductive success of respective ages. Expressing RferOR1 in *Drosophila* olfactory neurons shows that this receptor is narrowly tuned to the aggregation pheromone components ferrugineol and ferrugineone. Disrupting this gene using RNAi significantly reduces beetles’ responses to the aggregation pheromone [63]. Recently, genome data of the *R. ferrugineus* has become publicly available. It reports the complete gene family of the odorant receptors (80 genes) and the odorant binding protein (46 genes); however, the gustatory receptor genes have not been fully characterized [64].

This study aims to characterize the entire gustatory receptor gene family of the RPW using its genome and transcriptome data. Our findings provide essential information for future functional analysis, which will lead to a better understanding of how the RPWs discriminate plant hosts, locate appropriate egg-laying sites, and communicate via gustation.

## 2. Materials and Methods

### 2.1. R. ferrugineus Genetic Data

Our analysis used publicly available genetic data of the *R. ferrugineus* in the NCBI database. This included RNA sequence read archive (SRA) from four different tissues: adult male antenna (SRX3924074), adult female antenna (SRX3924089), adult male mouthparts (SRX3924065), and adult female mouthparts (SRX3924075). The RNA samples were isolated from beetles that originated in Thailand. RNA was sequenced using Illumina Hiseq2500 (paired-end read, 100 bp). For the genome data, we used a recently published genome (PRJNA524026) [64]. The genome was sequenced from a male beetle that originated from the United Arab Emirates using Illumina Hiseq, Illumina Novaseq, and Oxford Nanopore platforms and assembled into one genome with 108.0x coverage (782.1 Mb), consisting of 9 pseudochromosomes and an X chromosome [64].

### 2.2. Gene Identification

We first annotated the gustatory receptor genes from the transcriptome then extended the search to retrieve the full gene models and the entire gene family using genome data. Transcriptome analyses, including read quality controls, de novo assembly, and completeness assessment, were conducted using OmicsBox v2.0 (BioBam® Valencia, Spain). In brief, reads were trimmed and filtered to improve quality using Trimmomatic v0.38 [65]. Paired cleaned reads from the four libraries were assembled into a single transcriptome using Trinity v2.0.6 [66]. The completeness of assembly was assessed using BUSCO v4.1.2 [67]. We used Blast2GO v5.2.5 to annotate all the assembled transcripts using BlastX v2.12.0 against an insect non-redundant protein dataset from NCBI (https://ftp.ncbi.nlm.nih.gov/blast/db/) from Diptera and Coleopteran insects (E value = 10^−5^) (downloaded 30 November 2020). The expression level of each contig in each RNA sample was estimated using RSEM v1.3.3 [68]. Cleaned reads from each RNA sample were mapped into the assembled transcriptome contig then a mapped count table was created. Count per millions (CPM) was then converted to TMM (transcripts per kilobase million) to enable within-sample comparison.

We then constructed a local nucleotide database of the de novo assembled contigs on a PC using NCBI-blast-2.7.1+ program. The gustatory receptor proteins from a red flour beetle, *Tribolium castaneum*, [50], a mountain pine beetle (*Dendroctonus ponderosae*) [25], and a Colorado potato beetle (*Leptinotarsa decemlineata*) [24] were used as queries for the tBLASTn search (E value threshold = 10^−5^). DNA sequences of the significant BLAST hits were translated to protein using the ExPASy translate tool (https://web.expasy.org/translate/) (accessed on 1 December 2020). Translated proteins were aligned with gustatory receptor proteins of other beetles to check for conserved regions. Proteins without any conserved regions were discarded from a candidate gustatory receptor list.

To identify gustatory receptor genes from the genome data, we used a similar approach that we previously used to identify gustatory receptor genes from the *L. decemlineata* and *Plutella xylostella* genomes [24,69]. In brief, candidate GRs identified from the *R. ferrugineus* antennal and mouthparts transcriptome together with GRs from *T. castaneum*, *D. ponderosae*, and *L. decemlineata* were used as queries for a tBLASTn search against the *R. ferrugineus* genome using the NCBI BLAST tool v2.12.0 (E value threshold = 10^−5^). DNA sequences of the predicted gene regions were downloaded. Gene models (exon and intron regions) were predicted by comparing DNA sequences to closely related gustatory receptor proteins (from *R. ferrugineus* or other beetles) using GeneWise v2.4.1 [70]. Gene models were manually inspected by comparing their coding proteins to GRs identified from the transcriptome of other species. Protein translated from every exon in the gene model must show some degree of sequence conservation to previously identified gustatory receptors. Incorrect exons were fixed by selecting new candidate exon regions where the translated proteins showed conserved amino acids. The BLAST steps and annotation were repeated iteratively until no new gene models were found. Genomic location, protein length, cDNA, and protein sequences were reported. Protein topology was predicted using TOPCONS v2.0 [71].

### 2.3. Phylogenetic Analysis

A phylogenetic tree of the gustatory receptors from an RPW and other beetles were constructed from protein sequences using the maximum likelihood method. In brief, *R. ferrugineus*’ gustatory receptor proteins were aligned with the GRs from a red flour beetle (*T. castaneum*) [50], a mountain pine beetle (*D. ponderosae*) [25], and a Colorado potato beetle (*L. decemlineata*) [24] using MAFFT v7.0 [72]. Aligned sequences were trimmed to remove gappy or unaligned regions using trimAl v1.2 [73]. The adjusted alignment was used to construct a phylogenetic tree using PhyML v3.0 [74]. Branch supports were computed using the approximate likelihood-ratio test for branches (aLRT). The tree was visualized using FigTree v1.4.4.

## 3. Results

### 3.1. Antennal and Mouthpart Transcriptomes

#### 3.1.1. Transcriptome Data

The sequence reads are ~3.8–4.6 gigabases for each RNA sample (male antenna, female antenna, male mouthparts, and female mouthparts). The cleaned reads were assembled into a single transcriptome library, consisting of 181,024 contigs with an average length of 381.37 bp (Table 1). The completeness of the assembly was analyzed using BUSCO, which assesses the presence of single-copy or duplicated genes shared among selected taxa (in this case, insects). The *R. ferrugineus* antennal and mouthpart transcriptome matched 96.49% of the predefined orthologous groups, indicating a good representation of genes in our assembled transcriptome.

#### 3.1.2. Gene Annotation and Expression Level

**Figure 2:**
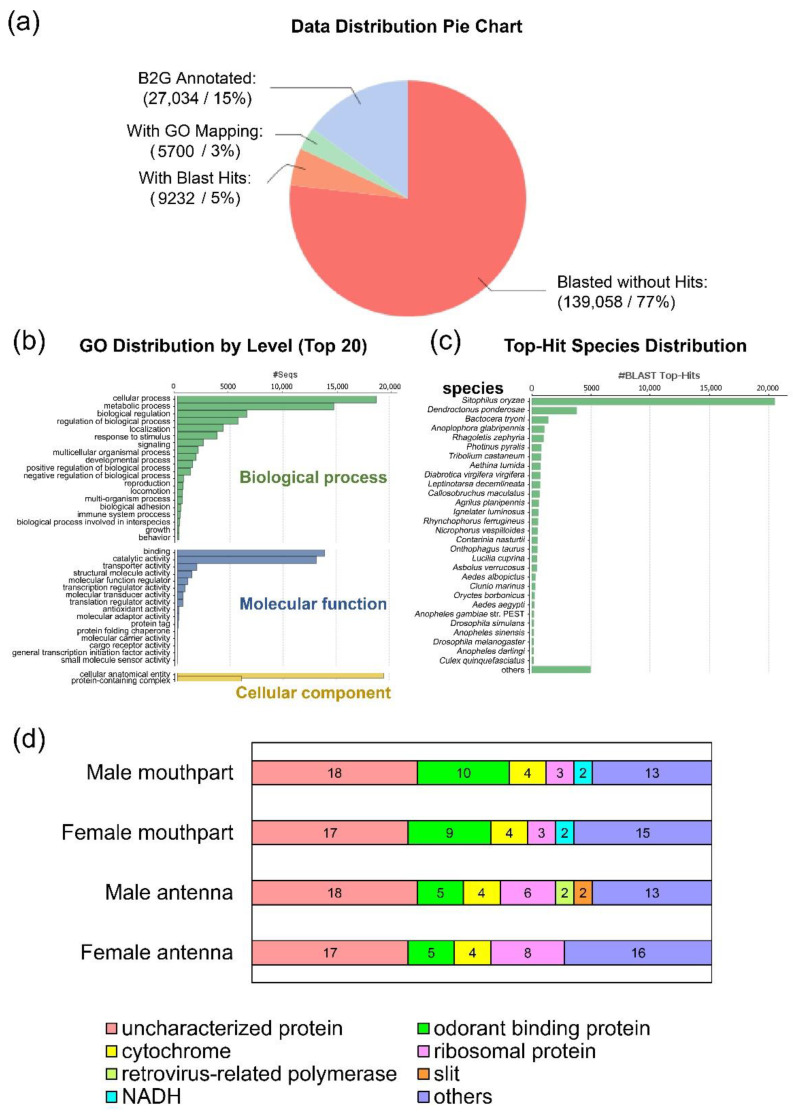
Antennal and mouthpart transcriptome data (**a**) Data distribution of the Blast2GO result (**b**) Gene ontology distribution by level (top 20) (**c**) Top-hit species distribution (**d**) Top 50 most expressed genes in the four transcriptomes. Genes are grouped into main classes. Genes that are not classified into a group are included in ‘others’.

Of the 181,024 contigs, 23% (41,916 contigs) retrieved blast hits (Figure 2a). The top 20 gene ontology (GO) is divided into biological process, molecular function, and cellular component. The most common GO terms for each category are cellular process, binding, and cellular anatomical entity, respectively (Figure 2b). Based on sequence similarity, the top BLAST hit species is the rice weevil, *Sitophilus oryzae*, followed by the mountain pine beetle, *Dendroctonus ponderosae* (Figure 2c). Both are beetles in the same family taxa of *R. ferrugineus* (Curculionidae).

We estimated the expression level of each contig by mapping cleaned reads to the assembled transcriptome and calculated the expression level in the TPM unit (transcripts per million). The expression level of transcripts within a transcriptome varies greatly. The expression levels of the top 50 most expressed genes of the four transcriptomes are in a similar range: male antenna (1217.84–141,595.42 TPM), female antenna (1371.28–177,092.54 TPM), male mouthparts (1945.09–120,919.68 TPM), and female mouthparts (2097.77–133,366.01 TPM). In this list, genes present in the four transcriptomes are odorant binding proteins, cytochrome, ribosomal protein, and some uncharacterized proteins (Figure 2d). The high expression of OBP genes in these organs correlates with the fact that both antenna and mouthparts of *R. ferrugineus* contain olfactory sensilla and serve as olfactory organs. In addition, OBPs are also expressed in the taste sensilla of various insects and are required for sensing tastants, particularly the bitter taste [75]. In the mouthpart transcriptome, genes encoding salivary protein and lysozyme are among the top 50 most expressed genes (class: ‘others’) for both males and females, reflecting the functional relevance of these genes. Full Blast2GO annotation results and expression level of transcripts are reported in the supplementary data (Table S1).

### 3.2. R. ferrugineus Gustatory Receptor Gene Family and Evolution

#### 3.2.1. Gustatory Receptor Gene Family

We first annotated gustatory receptor genes from the assembled transcriptome using the tBLASTn tool. This is the fastest way to get the first candidate set of *R. ferrugineus*’ gustatory receptor because protein sequences can be retrieved directly by translating assembled contigs which are intronless. Although we used both antenna and mouthparts from both sexes in the analysis, we could only identify 16 putative *Gr* genes, which is similar to previous studies that identified *Gr* genes from the whole body transcriptome (9 *Gr* genes) [58] and antennal transcriptome (15 *Gr* genes) [60]. Eight genes were full-gene models, and the remaining were missing either the N terminus, C terminus, or both. Based on sequence homology, there were four candidate CO_2_ receptors, two candidate sugar receptors, and ten candidate bitter receptors. The number of genes identified by this technique is much fewer than the number of *Gr* genes previously identified from other beetle genomes (60–234 genes) [24,25,26,50], suggesting that *R. ferrugineus* has many more genes to be identified from the genome.

We further extended the search using *R. ferrugineus* genome data. We have identified a total of 50 gustatory receptor genes encoding 65 gustatory receptor proteins via alternative splicing. Of these, 49 genes (98%) are full gene models, and only one gene model (*RferGr46*) has the C terminus missing. These proteins share a conserved motif ‘TYhhhhhQF’ (‘h’ = hydrophobic amino acid) near the end of the C terminus, which is a unique characteristic of insect gustatory receptors [76]. However, we note that there are mutations in this region in some GRs, e.g., ‘TY’ is replaced by ‘IY’ (RferGR4, RferGR5, RferGR33, RferGR34), ‘VY’ (RferGR38), ‘SY’ (RferGR31, RferGR32, RferGR39, RferGR49) and ‘QF’ are replaced by ‘QS’ (RferGR16, RfereGR23) and ‘QV’ (RferGR29, RferGR30). This is a common phenomenon in insect GRs [24,25,77]. Complete information, including protein and cDNA sequences of the *R. ferrugineus* gustatory receptors, is provided in Supplementary Table S2.

The *R. ferrugineus* gustatory receptors are 345–455 amino acids in length ($\bar{X}$ ± SE = 393.06 ± 3.42). Of the 62 full protein models, 47 proteins (76%) were predicted by TOPCONS to have the N terminus inside the cell and seven transmembrane domains, which are typical characteristics of insect gustatory receptors [28]. However, all nine sugar receptors (RferGR8-RferGR14, RferGR15a, RferGR15b) were predicted to have an N terminus outside the cell and eight transmembrane domains. We speculate that the atypical character of these sugar receptors is due to the miscalculation of TOPCONS, since BmGR8, a sugar receptor of the *Bombyx mori* moth that has been shown experimentally to have the N terminus inside the cell and seven transmembrane domains [28], was also predicted by TOPCONS to have the N terminus outside the cell and has eight transmembrane domains.

RferGRs are highly divergent. They commonly share less than 10% of their identity. However, some gustatory receptors share almost identical sequences, reflecting recent gene duplication. Some of them are located close to each other, e.g., *RferGr1* & *RferGr2* (~90,000 bp away), *RferGr4* & *RferGr5* (~150,000 bp away), and *RferGr33* & *RferGr34* (~200,000 bp away), whereas others are located millions of base pairs away, e.g., a cluster of *RferGr8-RferGr11* & *RferGr12-RferGr15* (~29 Million bp away) or even on different chromosomes, e.g., *RferGr37* & *RferGr49* (chromosome one and ten, respectively) and *RferGr42* & *RferGr44* (chromosome four and six, respectively). We argue that highly similar genes were not due to genome misassembly, where some genome sections are repeated due to erroneous. We compared the sequence identity of exons, introns, and intergenic regions between the highly similar genes. None of them showed 100% sequence identity in all three regions, which would be a sign of genome misassembly (Supplementary Table S3). Most of them have the highest identity in exons regions (98.33–100%), followed by the introns (95.63–100%), and the similarity drops to 43.99–99.88% in the intergenic regions, possibly due to relaxed purifying selection in non-coding regions. This confirms the presence of identified gustatory receptor genes in the *R. ferrugineus* genome and highlights the role of chromosomal duplication in expanding the gustatory receptor gene family in *R. ferrugineus*.

Six genes (*RferGr15*, *RferGr19*, *RferGr20*, *RferGr38*, *RferGr39*, *RferGr46*) are predicted to code for multiple proteins via alternative splicing (21 GRs, in total). This process increases the number of functional gustatory receptors in *R. ferrugineus* by 30%. *RferGr19* and *RferGr20* have the highest number of alternative splicing forms (a–f), which share the last exon (exon 2). Alternative splicing in the *Gr* genes is well-observed in other beetles. For example, *LdecGr48* (from *L. decemlineata*) and *TcasGr124* (from *T. castaneum*) encode for 13 and 24 functional GRs, respectively [24].

The identified *Gr* genes are supported by the expression in the transcriptome of chemosensory organs. We can detect an expression of 90% of the genes (45/50 genes) in either one of the four transcriptomes. However, these genes have very low expression levels, ranging from 0 to 21.894 TPM. We note that the expression levels of some of these genes cannot be measured accurately due to the highly conserved sequences between two genes. The low expression of *Gr* genes in the antennal transcriptome of *R. ferrugineus* was previously reported [60]. We could not detect the expression of *RferGr9*, *RferGr14*, *RferGr19*, *RferGr21,* or *RferGr22* in the four transcriptomes. These genes may be expressed at an undetectable level or expressed in specific conditions unexplored here, such as the larval stage or other organs (e.g., legs and ovipositors). However, its sequence homology and position in the phylogenetic tree confirm that they belong to this gene family.

#### 3.2.2. Phylogenetic Analysis

**Figure 3:**
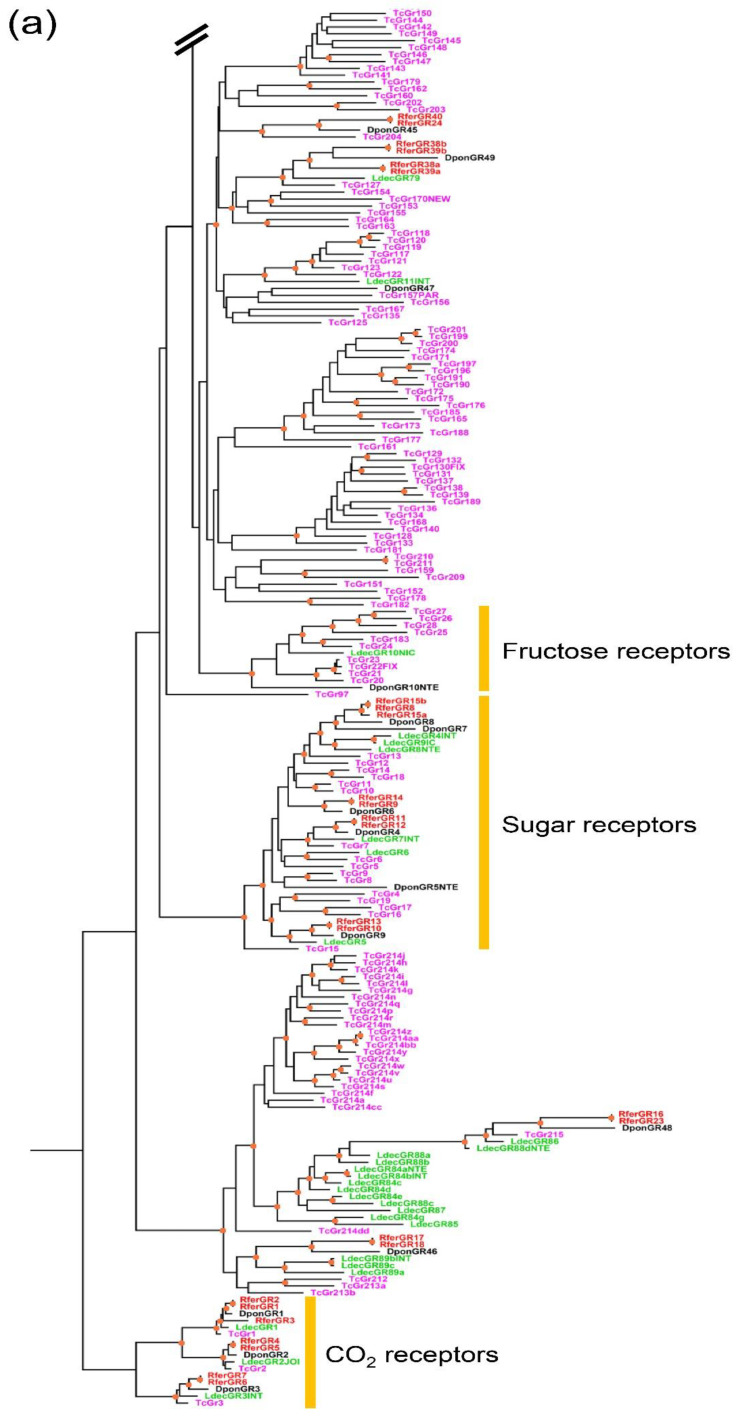

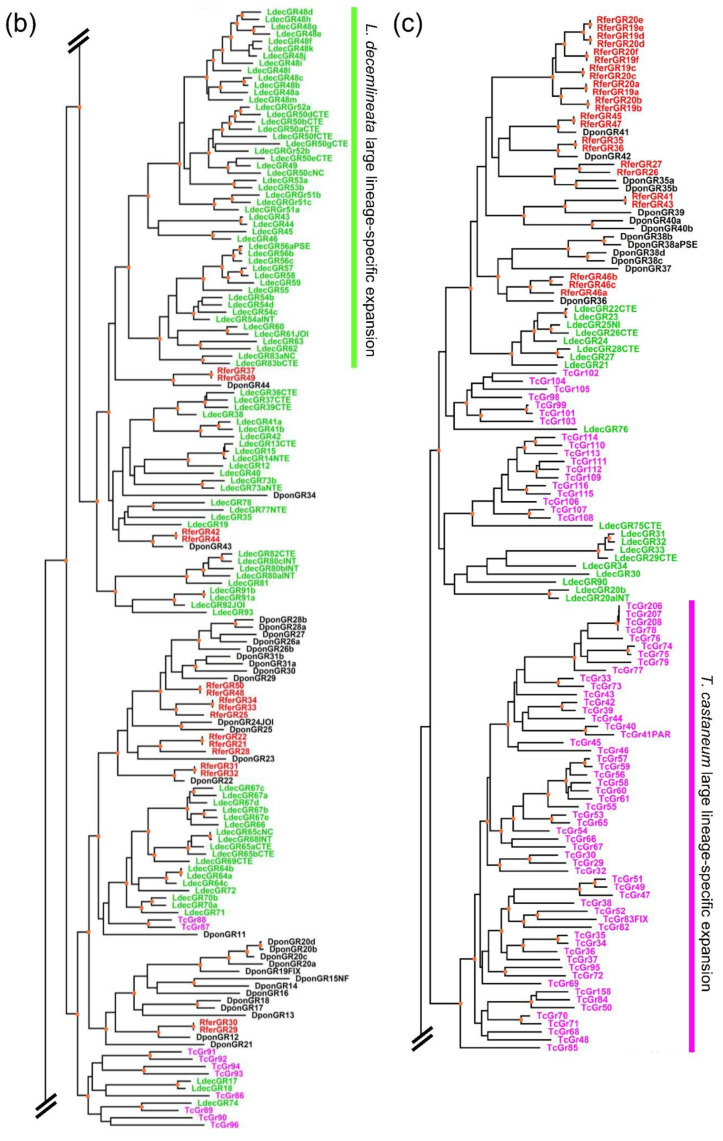
Phylogenetic tree of the red palm weevil and other pest beetle gustatory receptors. Due to its large size, tree was divided into three parts: (**a**) bottom, (**b**) middle, and (**c**) top. A tree was reconstructed from protein sequences using the maximum likelihood approach by PhyML3.0. The carbon dioxide, sugar, and fructose receptor clades were highlighted. Tree was rooted at the CO_2_ receptor clade. Branch supports were assessed using approximate likelihood-ratio test (aLRT); a value above 0.9 is shown with an orange dot on the node (Rfer = *Rhynchophorus ferrugineus*, Tcas = *Tribolium castaneum*, Dpon = *Dendroctonus ponderosae*, Ldec = *Leptinotarsa decemlineata*).

Phylogenetic relationships of gustatory receptors from *R. ferrugineus* and three other beetles reveal major gustatory receptor clades with different putative functions (Figure 3a–c). First, RferGR1-RferGR7 are members of the CO_2_ receptor clade (Figure 3a). This clade is the most conserved insect GR. In other species (*D. ponderosae*, *T. castaneum*, *L. decemlineata*), each has three CO_2_ receptors (GR1-3), and the orthologous relationship is a 1:1 ratio. We found gene duplication in all three CO_2_ receptors in *R. ferrugineus*: RferGR1-RferGR3 are the orthologs of GR1, RferGR4-RferGR5 the orthologs of GR2, and RferGR6-RferGR7 the orthologs of GR3. All of them have high branch supports (aLRT > 0.9).

RferGR8-15 are in the sweet receptor clade. Although this clade is relatively conserved, the orthologous relationship of genes in this clade is not a 1:1 ratio, and it exhibits some degree of species-specific expansion, particularly in *T. castaneum,* which has 16 genes (Figure 3a). The sugar receptors of *R. ferrugineus* are divided into two clusters of tandemly arrayed genes. The first cluster is RferGR8-RferGR11, and the second cluster is RferGR9-RferGR15. Proteins in these two clusters are direct paralogues (RferGR8 & RferGR15, RferGR9 & RferGR14, RferGR11 & RferGR13, and RferGR10 & RferGR13), which share almost 100% of protein identity. In the tree, these genes are sister taxa with very short branch lengths. The sugar receptors of *R. ferrugineus* are more closely related to *D. ponderosae* than other species. We did not detect the ortholog of a fructose receptor in the genome and transcriptome of *R. ferrugineus*. This gene is conserved in many insects; *L. decemlineata* and *D. ponderosae* have one ortholog, whereas *T. castaneum* has ten genes. This gene may be lost in the evolution of *R. ferrugineus* or missing due to the incompleteness of genome data. It is not likely that this gene in *R. ferrugineus* is as greatly expanded as in *T. castaneum*.

The remaining gustatory receptors are generally regarded as bitter receptors, accounting for a high proportion of the gene family. In *R. ferrugineus*, 49 gustatory receptors (75%) are in this group. Unlike CO_2_ and sugar receptors, bitter receptors do not have monophyletic relationships but are divided into many clades. The phylogenetic tree shows multiple lineage-specific expansion clades. Some are greatly expanded, e.g., the *L. decemlineata* specific-expansion clade (Figure 3b) and the *T. castaneum* specific-expansion clade (Figure 3c), having 49 and 56 receptors, respectively. Bitter receptors of *R. ferrugineus* are more closely related to *D. ponderosae* than other species.

#### 3.2.3. Chromosomal Location

**Figure 4:**
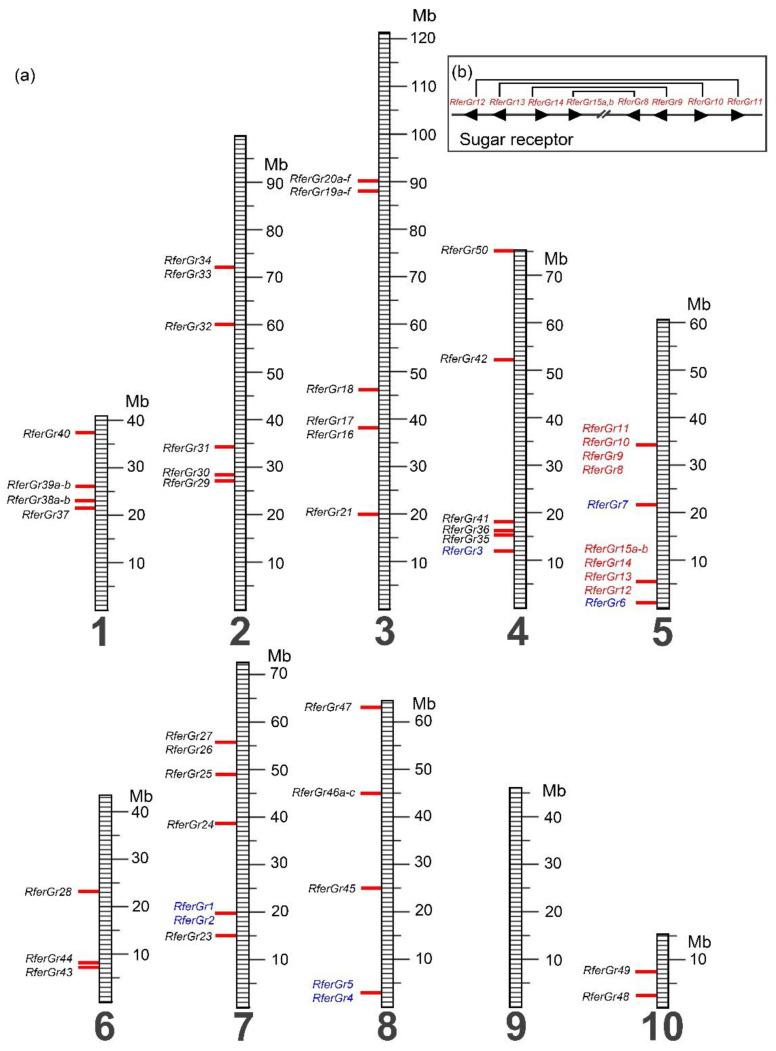
Chromosome mapping of the *Rhynchophorus ferrugineus* gustatory receptor genes (**a**) distribution of *RferGr* genes on the ten chromosomes (**b**) inverted duplication of the sugar receptor genes (Color code for *Gr* genes: blue = candidate c receptor, red = candidate sugar receptor, black = candidate bitter receptor).

Gustatory receptor genes are located on nine of the ten chromosomes of the RPW (it is absent on chromosome nine) (Figure 4a). Thirty-one genes (~60%) are found as a singleton (1 gene in a million base pairs region). Genes in the CO_2_ clade (*RferGr1-RferGr7*) are located on chromosomes four, five, seven, and eight. *RferGr1-RferGr2* and *RferGr4-RferGr5* are located adjacent to each other on chromosome seven and eight, respectively. Based on the high similarity of the DNA sequences, they might arise from recent gene duplication. Interestingly, all candidate sugar receptor genes (*RferGr8*-*RferGr15*) are located on chromosome five and divided into two clusters. The first cluster contains four genes, *RferGr8*-*RferGr11*, in the minus, minus, plus, and plus strand, respectively. Another cluster (*RferGr12*-*RferGr15*) is located about 24 million base pairs away (Figure 4b). Interestingly, genes in the two clusters are direct paralogous genes located in the same order but with inverted orientation, suggesting that inverted duplication and translocation play an important role in the evolution of the sugar receptors in *R. ferrugineus*. The DNA sequences of exons and introns of the paralogous genes are >95% identical, but the intergenic regions’ identity drops below 50% (Supplementary Table S3), suggesting that the duplication of this gene cluster occurred a long time ago, since there are many mutations in the intergenic regions. The fact that genes retain their sequence after duplication is possibly due to strong purifying selection. Other genes in the bitter clades are distributed in nine chromosomes. The distribution of genes in multiple chromosomes is a sign of an ancient gene family [76].

## 4. Discussion

The number of gustatory receptor genes in *R. ferrugineus* reported in this study (50 genes encoding 65 functional receptors) is higher than in previous studies, which identified genes from the transcriptomes (9–15 genes) [58,60]. A recent antennal transcriptome study in the American palm weevil, *Rhynchophorus palmarum*, has identified only seven gustatory receptor genes [78]. We believe that the transcriptome technique has many limitations in identifying *Gr* genes. First, *Gr* genes are expressed at very low levels, making some of them undetectable. In some cases, only partial sequences were obtained, making it hard to confirm that they are gustatory receptor genes. Second, the actual number of genes or functional receptors can be underestimated because some of *R. ferrugineus*’ gustatory receptor genes have almost identical sequences, and some of them have many alternative splicing forms. Third, genes may be expressed in the organs or developmental stages that are not studied. We believe that our analysis has identified most of the gustatory receptor genes from the *R. ferrugineus* genome, facilitating future comparative and functional studies. It is, however, possible that some *Gr* genes are missing due to the incomplete genome sequence or highly divergent genes. Different strains or populations of RPWs may also have a different number of genes. Further investigations when new *R. ferrugineus* genomes from different strains are available will help consolidate the number of genes in this species.

**Figure 5:**
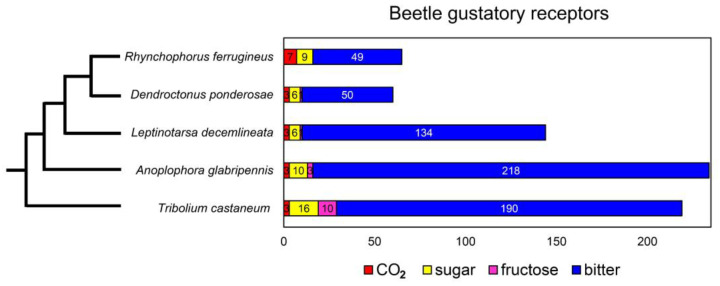
Comparative genomics of the gustatory receptor gene family of five pest beetles showing the number of genes for each putative function (CO_2_, sugar, fructose, and bitter receptors). The phylogenetic relationship of the five beetles was inferred from http://timetree.org (accessed on 20 May 2021). Sources of information: *D. ponderosae* [25], *L. decemlineata* [24], *A. glabripennis* [26], *T. castaneum* [50].

The number of functional GRs in *R. ferrugineus* is similar to *D. ponderosae* (60 GRs), but much fewer than *L. decemlineata* (144 GRs), *T. castaneum* (219 GRs), and *A. glabripennis* (234 GRs) [24,25,26,50] (Figure 5). Phylogenetic analysis shows that gustatory receptors from *R. ferrugineus* are more closely related to GRs from *D. ponderosae* than other species. Since the two beetles infest different host plants (palm plants vs. pine trees), close relationships of genes are primarily due to the close phylogenetic relationship of the two beetles (Curculionidae).

Both insect gustatory and odorant receptor genes are known for their highly dynamic evolution under the birth-and-death model [14,79]. Multiple gains and losses of chemoreceptor genes are observed in the evolution of insects, e.g., *Drosophila* spp., moths and butterflies, and ants [69,80,81]. Our study provides evidence of the dynamic change in the RPW *Gr* gene family, including recent gene duplication of CO_2_ receptor genes and the duplication of a sugar receptor gene cluster on chromosome five This reflects how insects adapt to a wide range of chemicals related to their critical ecological success, such as plant hosts, egg-laying sites, and chemical communication. Although RPW is regarded as a generalist species, its preferred hosts are palm plants, including 26 palm species belonging to 16 different genera [82]. The relatively small number of *Gr* genes in *R. ferrugineus* supports the previous observation that stenophagous beetles are more likely to have a reduced number of chemosensory-related genes than species that can feed on various plant hosts [25].

*R. ferrugineus* has more CO_2_ receptors (seven genes) than other beetles (three genes) (Figure 5). Perception of CO_2_ is essential in beetles; for example, larvae of the giant rhinoceros beetle *Trypoxylus dichotomus* are attracted to CO_2_, a by-product of fermented humus that the larvae feed on [83]. It is still unclear how CO_2_ contributes to the RPW’s gustation. It might be a cue from a damaged tree that allows female beetles to locate egg-laying sites or a cue from aggregating beetles where mating occurs. *R. ferrugineus* has nine putative sugar receptors in the same range as other beetles (6–16 receptors) (Figure 5). The roles of sugar receptors in the ecological success of RPWs are unknown, but they are presumably involved in detecting nutrient-rich foods, since adult beetles feed on some fruits, e.g., banana (Figure 1a), date palm, pineapple, and sugarcane. In *Drosophila*, sugar receptors can be inhibited by bitter tastants, thus functioning as a dual sensor that helps discriminating foods [84]. Finally, *R. ferrugineus* has 49 putative bitter receptors, about the same number as *D. ponderosae* but 3–4 times fewer than *L. decemlineata*, *A. glabripennis*, and *T. castaneum* (Figure 5). It is generally accepted that these receptors help insects avoid toxic plant chemicals, and some ligand–receptor relationships have been reported [27,85]. For example, *Drosophila* GR28b is essential for detecting saponin, the antifeedant and insecticide from soapbark, *Quillaja saponaria*. Mutant flies have trouble avoiding this chemical [86]. The bitter receptor, PxylGR34, of a diamondback moth, *Plutella xylostella*, is tuned to the plant hormone brassinolide, which inhibits larval feeding and female oviposition [87]. Further studies are required to elucidate the significance of bitter receptors to the biology of the RPWs.

RPWs cause severe economic loss in palm plant production globally [1]. Due to their highly invasive nature, the FAO has called for international collaboration and a multidisciplinary strategy to control and stop the spreading of this pest [4]. The molecular basis of its chemoreception has been a center of research attention because of its potential for improving pheromone/kairomone bait and biosensors that help the early detection of this pest and mass trapping. Previous research included the identification of chemosensory-related genes [58,59,60,64], functional analysis of odorant binding protein and a conserved olfactory co-receptor [62], and recently identification of the aggregation pheromone receptor [63]. Our study contributes to the understanding of the complex nature of this beetle’s chemosensory function by characterizing all the gustatory receptor genes used for gustation—an under-researched area. Our findings provide a platform for future comparative study and functional analysis of these gustatory receptors.

## 5. Conclusions

We comprehensively characterized the gustatory gene family of the red palm weevils, *R. ferrugineus,* using its genome and transcriptome data. There are 50 genes encoding 65 functional gustatory receptors via alternative splicing. These receptors include 7 CO_2_ receptors, 9 sugar receptors, and 49 bitter receptors. Our results provide fundamental knowledge of the molecular basis of gustation, which is a crucial sense for locating and discriminating appropriate plant hosts, egg-laying sites, and possibly for chemical communication. Our study facilitates future functional analysis of these receptors and could improve control methods for this highly destructive pest.

## Figures and Tables

**Table 1:** Transcriptome statistics. RNA was sequenced using Illumina Hiseq2500 platform (paired-end reads, 100 bp), and reads were de novo assembled using Trinity in OmicsBox (BioBam).

Libraries	Details	Numbers
Male antenna	Total reads	42,872,182
	%GC	38
Female antenna	Total reads	38,301,355
	%GC	36
Male mouthparts	Total reads	40,701,754
	%GC	38
Female mouthparts	Total reads	45,615,562
	%GC	37
Pooled assembly	Min contig length	101
	Max contig length	27,357
	N50	953
	Mean contig length	381.73
	Total bases (bp)	69,037,346
	Unique contigs (Unigenes)	181,024

## Data Availability

The data presented in this study are available in the Supplementary Materials section.

## Supplementary Materials

The following are available online at https://www.mdpi.com/article/10.3390/insects12070611/s1, Table S1: BLAST2GO results and estimated expression level of transcripts in the four transcriptomes, Table S2: Full information of the red palm weevil, *Rhynchophours ferrugineus*, gustatory receptor genes (completeness of gene model, protein length, predicted topology by TOPCONS (N terminus inside or outside cell, and number of transmembrane domains), conserved motif near the C terminus, chromosomal location, matched transcripts in a transcriptome, estimated expression level, protein, and cDNA sequence), Table S3: Percent identity between closely related gustatory receptor genes including protein sequence, exons, introns, and intergenic region (5000 bp downstream unless stated otherwise in Note).
